# Role of Luteolin as Potential New Therapeutic Option for Patients with Glioblastoma through Regulation of Sphingolipid Rheostat

**DOI:** 10.3390/ijms25010130

**Published:** 2023-12-21

**Authors:** Stefania Elena Navone, Laura Guarnaccia, Massimiliano D. Rizzaro, Laura Begani, Emanuela Barilla, Giovanni Alotta, Emanuele Garzia, Manuela Caroli, Antonella Ampollini, Aniello Violetti, Noreen Gervasi, Rolando Campanella, Laura Riboni, Marco Locatelli, Giovanni Marfia

**Affiliations:** 1 Laboratory of Experimental Neurosurgery and Cell Therapy, Neurosurgery Unit, Foundation IRCCS Ca’ Granda Ospedale Maggiore Policlinico, 20122 Milan, Italy; stefania.navone@policlinico.mi.it (S.E.N.); laura.guarnaccia@policlinico.mi.it (L.G.); massimilianodomenico.rizzaro@studenti.unimi.it (M.D.R.); laura.begani@policlinico.mi.it (L.B.); manuela.caroli@policlinico.mi.it (M.C.); antonella.ampollini@policlinico.mi.it (A.A.); marco.locatelli@policlinico.mi.it (M.L.); 2 Andremacon Biotech Srl, Viale Ortles, 22/4, 20141 Milan, Italy; e.barilla@andremacon.com (E.B.); g.alotta@andremacon.com (G.A.); campanella.rolando@gmail.com (R.C.); l.riboni@andremacon.com (L.R.); 3 Reproductive Medicine Unit, Department of Mother and Child, San Paolo Hospital Medical School, ASST Santi Paolo e Carlo, 20142 Milan, Italy; emanuele.garzia@asst-santipaolocarlo.it; 4 Aerospace Medicine Institute “A. Mosso”, Italian Air Force, 20138 Milan, Italy; 5 Space Attache’, Embassy of Italy in Washington DC, Washington, DC 20008, USA; aniello.violetti@esteri.it; 6 Alcamena Stem Cell Therapeutics, 1450 South Rolling Road, Suite 4.069, Halethorpe, MD 21227, USA; noreen.gervasi@gmail.com; 7 Department of Medical-Surgical Physiopathology and Transplantation, University of Milan, 20122 Milan, Italy

**Keywords:** glioblastoma stem cells, luteolin, sphingosine-1-phosphate, ceramide, sphingolipid rheostat, temozolomide, glioma stem cells

## Abstract

Glioblastoma (GBM) is the most aggressive brain tumor, still considered incurable. In this study, conducted on primary GBM stem cells (GSCs), specifically selected as the most therapy-resistant, we examined the efficacy of luteolin, a natural flavonoid, as an anti-tumoral compound. Luteolin is known to impact the sphingolipid rheostat, a pathway regulated by the proliferative sphingosine-1-phosphate (S1P) and the proapoptotic ceramide (Cer), and implicated in numerous oncopromoter biological processes. Here, we report that luteolin is able to inhibit the expression of SphK1/2, the two kinases implicated in S1P formation, and to increase the expression of both SGPL1, the lyase responsible for S1P degradation, and CERS1, the ceramide synthase 1, thus shifting the balance toward the production of ceramide. In addition, luteolin proved to decrease the expression of protumoral signaling as MAPK, RAS/MEK/ERK and PI3K/AKT/mTOR and cyclins involved in cell cycle progression. In parallel, luteolin succeeded in upregulation of proapoptotic mediators as caspases and Bcl-2 family and cell cycle controllers as p53 and p27. Furthermore, luteolin determined the shutdown of autophagy contributing to cell survival. Overall, our data support the use of luteolin as add-on therapy, having demonstrated a good ability in impairing GSC viability and survival and increasing cell sensitivity to TMZ.

## 1. Introduction

Glioblastoma *IDH*-wildtype (herein called GBM) is the most common malignant brain tumor and represents more than 60% of gliomas in adults [1]. Along with oligodendroglioma and ependymoma, it belongs to the class of gliomas and is the most aggressive grade among astrocytomas (grade IV) according to WHO 2021 classification [2]. Despite advances in the field of neuro-oncology, GBM is still considered an incurable tumor, with a mean overall survival (OS) not exceeding 15 months [3]. In operable patients, radical resection is the therapeutic strategy of first choice and the most important prognostic factor [4]. Surgery is almost always followed by adjuvant radiotherapy combined with concomitant chemotherapy following the Stupp protocol, consisting of the administration of low-dose temozolomide (TMZ) (75 mg/m^2^ of body surface area) for 45 days associated with fractionated radiotherapy with 2 Gy per session for 6 weeks for a total of 60 Gy. With the Stupp protocol, patients achieved an advantage of 2-month longer median survival compared with the arm treated with radiotherapy alone, and the two-year survival rate was 26.5% with radiotherapy plus TMZ and 10.4% with radiotherapy alone [5]. TMZ should prove its major efficacy on patient presenting an hypermethylation of O^6^-methylguanine (O^6^-MeG)-DNA methyltransferase (*MGMT*) promoter, with an indication of 9% methylation as cut-off [6]. However, after an initial response, unfortunately about 90% of patients relapse and acquire resistance to chemotherapy [3]. Numerous therapies have been proposed for the treatment of recurrent GBM, such as carmustine wafer [7], bevacizumab [8] and tumor treating fields [9]. However, none of these have been able to achieve clinically meaningful results. The reasons why GBM is so difficult to treat lie in its biological complexity and heterogeneity, both at a cellular and molecular level. The strong ability to infiltrate the surrounding tissue makes total surgical removal almost impossible, and the presence of cell niches composed of glioblastoma stem cells (GSCs) within the tumor mass allows GBM self-maintenance and the ability to reform following therapy [10].

Luteolin (3,4,5,7-tetrahydroxy flavone) is a molecule belonging to the class of flavonoids. It is a phytochemical contained in numerous foods including celery, sweet bell peppers, carrots, onion leaves, broccoli and parsley [11]. The study of naturally occurring compounds began a few years ago, from the time when the phenomenon of oxidative stress began to take hold as the pathogenic basis of certain types of diseases, such as cancers and inflammatory diseases. Especially, luteolin has been analyzed in research laboratories as a potential antineoplastic drug for more than twenty years. 

In GBM, the antioncogenic perspective of luteolin has been correlated with its capacity to inhibit cell growth, to induce apoptosis, to slow down and reduce invasion and migration, and to downregulate iNOS expression, proved on human glioblastoma T98G (mutant p53) and U87MG (wild-type p53) cancer cell lines [12].

In this study, we analyzed the role of luteolin as a potential therapeutic adjuvant option for the treatment of GBM, investigating the luteolin-mediated modulation of the sphingolipid rheostat, actioned by the proapoptotic ceramide (Cer) and the protumoral sphingosine-1-phosphate (S1P). S1P is a bioactive lipid with pleiotropic functions able to stimulate cellular processes strictly related to cancer, such as proliferation, invasion, survival and angiogenesis [13]. S1P is produced via the conversion of ceramide (Cer) to sphingosine (Sph), which is then phosphorylated to S1P by Sph kinases (SphK1/2). A perturbation of sphingolipid reosthat in favor of S1P represents an anti-apoptotic, pro-survival stimulus exploited by many tumors to self-maintain and escape therapies. 

We previously reported that GBM also exhibits high concentrations of S1P in tumor tissue, as well as an increased expression of SphK1/2 compared with normal tissues [14]. At the same time, the enzyme Ceramide synthase 1 (*CERS1*), deputed to synthesize ceramide, proved to be downregulated in GBM. In this context, we found that luteolin acts by inhibiting SphK2 activity in colorectal cancer, significantly decreasing S1P levels and increasing Cer synthesis, overall resulting in reduced viability of cancer cells [14].

From these premises, here we report for the first time the efficacy of luteolin in decreasing cell viability and survival of resistant patient-derived GSCs, with a molecular mechanism associable with the inhibition of S1P signaling the potentiality of luteolin as a natural add-on therapy for GBM patients. 

## 2. Results

### 2.1. Study Cohort

In order to characterize and test patient-derived GSCs resistant to TMZ (rGSCs), we analyzed the *MGMT* promoter methylation status of GBMs by pyrosequencing technique and classified samples based on the cut-off of 9%, as a value > 9% is considered a favorable prognostic indicator, associated with a better response to TMZ [6].

Therefore, we selected a cohort of hypomethylated n = 10 GSCs, considered resistant GSCs, with a mean *MGMT* promoter methylation of 4% ± 1.6. The mean age of patients was 73.8 ± 10.85 years and 80% of patients died after a mean course of 6.2 months after surgery. The mean mitotic index analyzed with Ki67 was 21.8 ± 15.4. All tumors analyzed had no IDH gene mutations.

### 2.2. Cell Culture Challenging

With the aim of confirming the maintenance of the molecular signature in cultured GSCs, pyrosequencing analysis were exploited to measure MGMT promoter methylation at different culture passages, with no significant differences reported.

**Figure 1:**
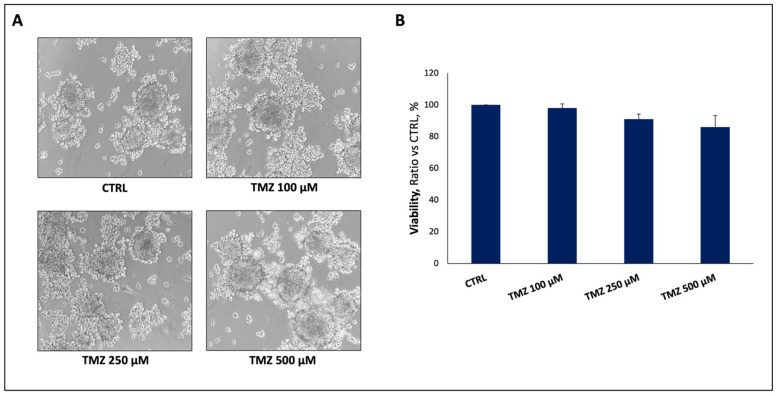
(**A**) Representative images of GSC challenged with increasing doses of TMZ, reporting a dose-independent chemotherapy resistance. (**B**) Viability data of GSC treated with increasing doses of TMZ. No statistical significance was reported in the treatment groups compared to control. Data are the means ± standard deviation of at least three experiments, run in triplicate. Magnification 10×.

To prove the effective TMZ resistance, GSCs were challenged with increasing dose of TMZ, until reaching a dose comparable to the therapeutic one and already known to be able to determine cell death (range 100–500 μM). This experiment, presented by representative cell culture images in Figure 1A, demonstrated no cell response to TMZ, even at the highest dose, which affected only 15% of cell viability (Figure 1B). 

**Figure 2:**
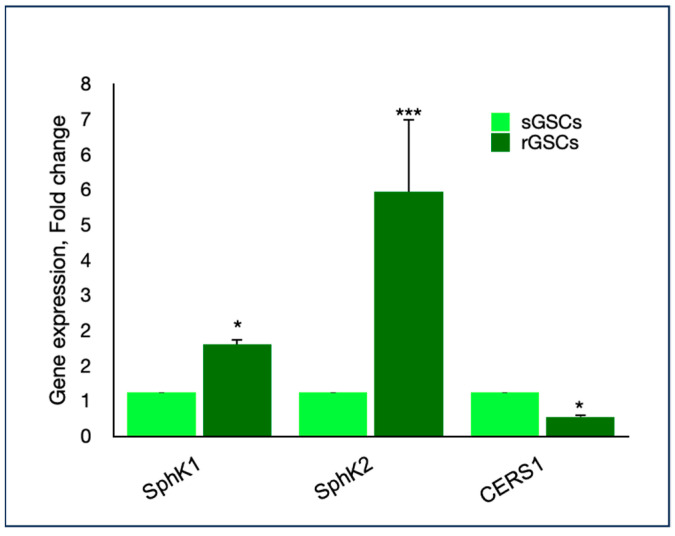
Gene expression analysis conducted on sensitive and resistant GSCs (sGSCS and rGSCs), proving a significant upregulation of *SphKs* and a downregulation of *CERS1*. Data are the means ± standard deviation of at least three experiments, run in triplicate. * *p* < 0.05, *** *p* < 0.001.

Introducing the potential contribution of sphingolipid rheostat in TMZ-resistance, rGSCs exhibited a two-fold increased expression of *SphK*1 and a five-fold upregulation of *Sphk*2, together with the parallel downregulation of *CERS1*, each compared with TMZ-sensitive GSCs (sGSCs), previously isolated and used as control (Figure 2).

After this characterization, we started testing rGSCs which, since they did not show any decay from treatment with TMZ, corroborated the urgent need of novel targeted treatments.

In this view, given their differential expression in sGSCs and rGSCs, we considered SphK targeting a promising approach to affect rGSCs (hereafter called GSCs) viability and survival.

**Figure 3:**
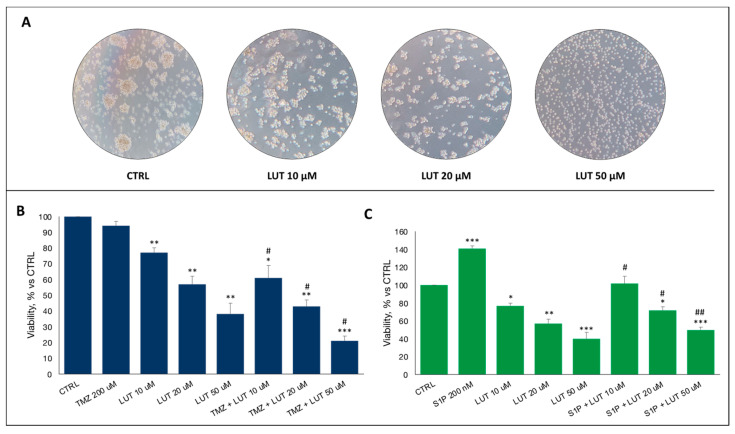
Effect of luteolin on GSCs. (**A**) Representative images of GSCs treated with increasing doses of luteolin, showing morphologic changes in neurosphere conformation. (**B**) Viability assay demonstrated a decrease of viable cell percentage after treatment with luteolin, alone or in combination with TMZ, compared to CTRL. (**C**) The same effect was observed also in co-administration with S1P, as proliferative stimulus. Data are the mean ± standard deviation of at least three experiments, run in triplicate. Magnification 4×. * statistical significance of treatment vs. CTRL, # statistical significance of combined treatment vs. single treatment. * *p* < 0.05, ** *p* < 0.01, *** *p* < 0.001. # *p* < 0.05, ## *p* < 0.01.

As supposed, treatment with increasing concentration of luteolin (10, 20 and 50 μM), resulted in clear cell distress, loss of neurosphere morphology typical of GSCs and a decrease in the percentage of viability to 77%, 57% and 38%, respectively (Figure 3A,B). Surprisingly, the combined treatment with different doses of luteolin and TMZ showed a synergic efficacy in decreasing GSC viability, even considering the intrinsic resistance, to 61%, 43% and 21%, respectively (Figure 3B).

Further proof of the effectiveness of luteolin came from the association with S1P, its agonist and key factor in the proliferation of tumor stem cells, the single administration of which caused an increase in proliferation of about 40%, which was, however, inhibited by the concomitant administration of luteolin (Figure 3C). Already at the lowest concentration of 10 μm, luteolin is able to counteract S1P oncopromoter signal, mimicking a possible contrast mechanism of the increase of S1P in the tumor microenvironment.

**Figure 4:**
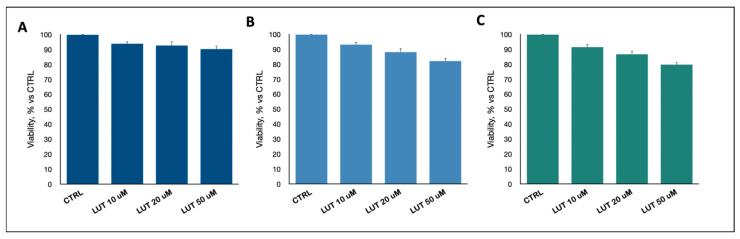
Effect of increasing doses of luteolin on astrocytes (**A**), NPC (**B**) and NPSC (**C**). No significant decreases in cell viability were observed, not even at the highest dose tested. Data are the mean ± standard deviation of at least three experiments, run in triplicate.

Notably, the same concentration of luteolin was administered to a healthy brain, derived as astrocytes (Figure 4A), neuronal primary cell (NPC, Figure 4B) and neural progenitor stem cells (NPSC, Figure 4C), in order to evaluate the detrimental toxicity of this compound on the normal brain counterparts. Interestingly enough, luteolin did not affect the viability of these cells, confirming its specifically targeting activity. 

**Figure 5:**
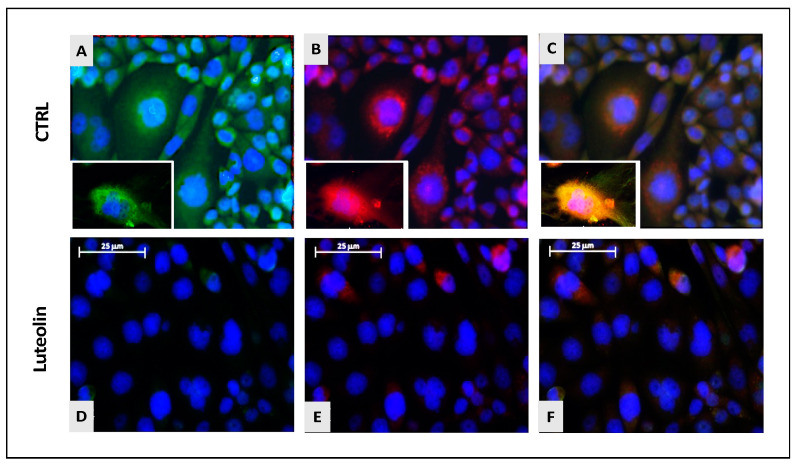
Expression of SphK1 (**A**, green labeling) and SphK2 (**B**, red labeling) merged with nuclear marking with DAPI (blue labeling) (**C**) in untreated GSCs (**A**–**C**) and GSC treated with luteolin 50 μM (**D**–**F**). In C and F (merging), the arrangement of the two kinases within the cells is appreciable. Larger magnifications of CTRL GSCs available in white boxes. SphK2 appears to assume a perinuclear position, while SphK1 appears to be positioned at the membrane level. Magnification 20×. Scale bar: 25 μm.

To confirm the influence of luteolin on sphingolipid rheostat, we performed immunofluorescence analysis focusing on SphK1/2, widely recognized as luteolin molecular targets. Interestingly, GSCs treated with luteolin 50 μM, identified as the optimal dose, showed a weaker labeling for both SphK1 and SphK2, compared to control, suggesting a downexpression of the kinases responsible for S1P production and downstream protumoral effects (Figure 5).

**Figure 6:**
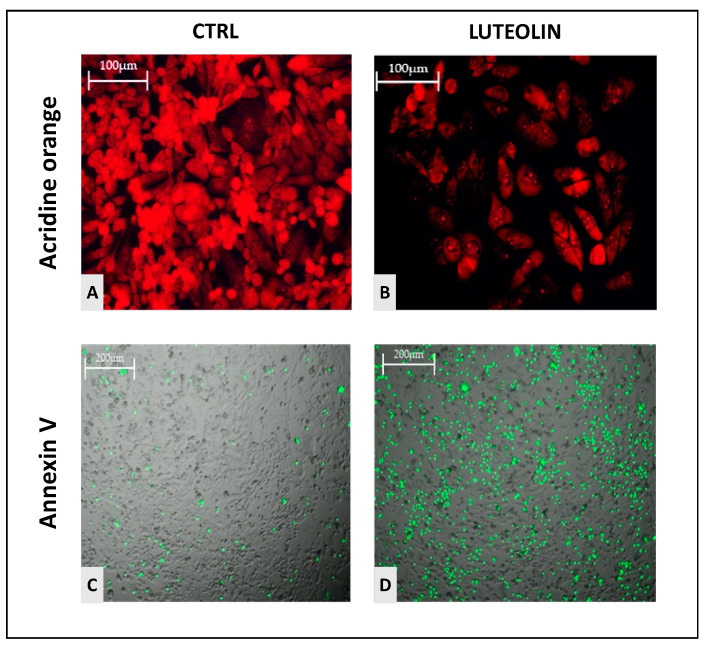
Marking cellular autolysosomes destined for autophagy (bright red) with acridine orange in cells untreated (**A**) and treated with luteolin μM (**B**). Degree of apoptosis (green dots) detected in cells untreated (**C**) and treated with luteolin μM (**D**), with Annexin V protocol. Scale bar: 100 μM (**A**,**B**) and 200 μM (**C**,**D**).

Through acridine orange, the ability of GBM cells to trigger autophagy mechanisms was examined. Autophagy is, in fact, a way exploited by cancer cells to “recycle” cytoplasmic material into nutrients directed to their own sustenance, being considered a tumor mechanism of survival. In Figure 5, the signal in bright red marks autophagic autolysosomes. The signal is much more intense in control cells (Figure 6A) and strongly decreases, almost disappearing in luteolin-treated cells (Figure 6B).

Furthermore, the degree of treatment-induced apoptosis was explored by measuring phosphatidylserine exposure on the outer side of the plasma and vesicular membrane by Annexin V assay. This biological mechanism is displayed by the increased luminescence (green dots) in GSCs treated with luteolin (Figure 6D), compared to control (Figure 6C). 

**Figure 7:**
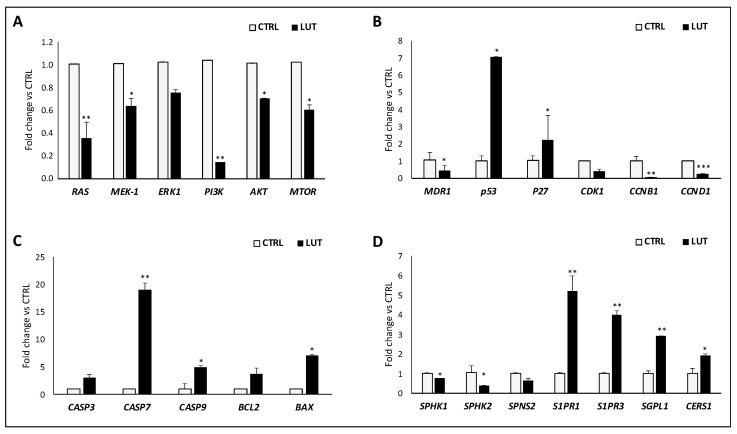
Level of change in gene expression of proliferative (**A**), cell cycle regulation (**B**), apoptosis (**C**) and sphingolipid-metabolism and signaling mediators (**D**). Data are presented as fold change versus CTRL. * *p* < 0.05, ** *p* < 0.01, *** *p* < 0.001.

Finally, the potential molecular mechanism underlying luteolin efficacy in decreasing GSC viability, resistance and survival was investigated by gene expression analysis with RealTime-PCR. Data reported in Figure 7 show an interesting downregulation of MAPK signaling *RAS/MEK/ERK* and *PI3K/Akt/mTOR* (Figure 7A), in parallel with the downexpression of cyclins influencing cell cycle progression and its arrest and the upregulation of cell cycle regulators as *p53* and *p27* (Figure 7B).

The proapoptotic induction by luteolin was confirmed by the overexpression of caspases-3, -7 and -9, as well as those of *Bcl-2* and *Bax*, whose ratio resulted in favor of the proapoptotic Bax (Figure 7C).

Finally, our driving hypothesis about the modulator effects of luteolin on sphingolipid rheostat and signaling, converged on the downregulation of both *SphK1* and *SphK2*, accompanied by a downregulation of *SPNS2*, the specific transporter of S1P outside cell membrane. In parallel, the enzyme responsible to shift the balance toward cell apoptosis, sphingosine-1-phosphate lyase 1 (*SGPL1*), involved in sphingolipid catabolism and the Ceramide Synthase 1 (*CERS1*), the enzyme that catalyzes the synthesis of ceramide were all upregulated by luteolin treatment (Figure 7D). 

**Figure 8:**
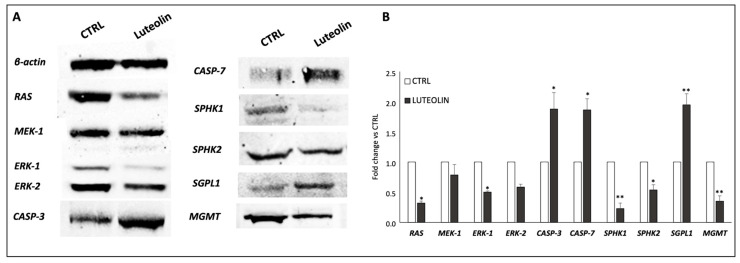
Protein expression levels of representative mediators of proliferation, apoptosis and sphingolipid rheostat. (**A**) Representative images of Western Blot analysis and relative quantification performed by ImageJ (Rasband, W.S., ImageJ, U. S. National Institutes of Health, Bethesda, Maryland, USA, https://imagej.net/ij/, 1997–2018) (**B**). Data have been normalized to β-actin expression and presented as fold change versus untreated CTRL. * *p* < 0.05, ** *p* < 0.01.

Gene expression data were confirmed by the measurement of protein levels (Figure 8), as Western Blot revealed a downregulation of RAS/MEK/ERK1-2, as well as an upregulation of Caspase-3 and -7. Regarding sphingolipid rheostat, we reported a diminished expression of SPHK1/2 also at protein level, together with the increase of SGPL1. Interestingly enough, we tested the expression of MGMT to explore the possibility of a decreasing effect of luteolin over hypomethylated cells, discovering a significant downregulation potentially meaning a restoring of sensitivity to TMZ.

## 3. Discussion

GBM is the most lethal brain tumor in adults, representing an extreme therapeutic challenge. The urgent need to discover novel paradigms of treatment shifts the view of research community toward a model of precision medicine, prompting to identify novel targets and molecules. In the last decades, the idea to use natural components to be placed side to side with classic chemotherapeutic agents has quickly caught on, thanks to the wide distribution within foods, the bioavailability, the ease of extraction and the near absence of adverse side effects when opportunely administered. 

In this context, in our study we tested the anti-tumoral effects of luteolin, a naturally occurring secondary metabolite belonging to the class of flavones, identified as one of the candidate bioactive constituents that potentially contribute to the preventive and pharmacological values of several medicinal plant species. Luteolin has been described as an effector in multiple biological processes, being anti-oxidant, anti-inflammatory, anti-allergy, anti-diabetics, neuroprotective and anti-cancer [15].

The antitumoral activity of luteolin has been correlated to its action in inhibiting tumor cell invasion, metastasis, proliferation and angiogenic-related mechanisms. The molecular events underlying these effects consist of promotion of apoptosis, regulation of the cell cycle, suppression of kinases and diminution of transcription factor activity [16]. Notably, luteolin has been reported to exert an inhibitory effect on tumor cell proliferation with IC50 values in the range between 3–50 µM in vitro [17].

This evidence inspired the potential use of luteolin against GBM and encouraged the testing of luteolin on a peculiar GBM cell subpopulation, known to be the most aggressive and therapy-resistant, the GSCs, responsible for tumor onset, maintenance, and recurrence. To be more selective with the tumor model, we screened GSCs with a very low level of methylation of the MGMT promoter (about 4%), known to be a negative prognostic factor for patients diagnosed with GBM. Furthermore, we challenged GSCs with increasing doses of TMZ, to be sure to select and test authentic therapy-resistant tumor stem cells. 

The administration of increasing doses of TMZ confirmed the high therapy-resistance of GSCs, as, also at the highest concentration, TMZ did not significantly impact cell viability, and neurospheres did not lose their typical aggressive phenotype. 

Taking advantage of our previous research in the field, we explored the possible contribution of sphingolipid rheostat in cell sensitivity to chemotherapy, with the aim of identifying a potential targetable pathway. 

Sphingolipids are a family of lipid signaling molecules that play pleiotropic effects in cellular regulation. The two primary metabolites, S1P and Cer, maintain rheostat balance and play opposing roles, controlling cell fate. S1P is able to stimulate cellular processes strictly related to cancer, such as proliferation, invasion, survival and angiogenesis, while Cer favors anti-proliferative and cell death pathways, such as senescence and apoptosis. A shift in this balance toward S1P results in tumor cell survival and resistance to chemotherapies. In this context, we previously demonstrated that S1P plays pleiotropic functions in gliomagenesis [18]. For example, we reported that fast proliferating GSCs, compared to slow-proliferating GSCs, convert faster Sphingosine to S1P, rapid degrade newly synthesized Cer, and release a 10-fold higher amount of S1P in the extracellular milieu [18].

With the aim of exploring if sphingolipid rheostat could be involved in TMZ resistance, we evaluated gene expression in rGSC and sGSC, discovering a shift in the balance toward the production of S1P, through the overexpression of kinase catalyzing its production, SphK1/2, in parallel with the downregulation of the ceramide synthase. Therefore, it appeared reasonable that targeting sphingolipid metabolism and signaling to shift the rheostat in favor of apoptotic ceramide, deserves consideration as a novel therapeutic strategy to fight aggressive cancers as GBM. 

Interestingly, our previous studies demonstrated that luteolin acts by suppressing ceramide generation and inhibiting the activation of Akt and SphK2, with the consequent reduction of S1P, an activator of Akt [14,19]. 

From these premises, we started challenging GSCs with increasing dose of luteolin, alone or in combination with TMZ and S1P to examine the synergic or antagonist effect on cells viability and functionality.

First of all, our data demonstrated that luteolin exerts a therapeutic-like dose-dependent effect on GSCs, with viability drastically decreasing up to 40% at the highest concentration (50 μM), in line with scientific literature on other less aggressive human cancers. In addition, the combination of luteolin with TMZ, achieved a very promising result, reaching the lowest viability observed in this study (about 20–25%). Therefore, luteolin proved to be able to increase the cytotoxicity of TMZ, whose single administration failed to significantly impact cell viability, probably due to the *MGMT* hypomethylation of tested GBMs.

This synergy may represent an unexplored way to overcome the therapy escape mechanism of resistant tumor stem cells, opening up possible new avenues for the treatment of relapse.

Furthermore, luteolin proved to be able to override the proliferative pulse of S1P, as if the administration of S1P determined an increase of cell viability up to 150%, the combined treatment with luteolin reported cell viability at a level comparable to control at the lowest dose (10 μM) and to a reduction up to 50% at the highest (10 μM), neutralizing S1P.

This effect was accompanied by the downregulation of SphK1 and SphK2, the kinases responsible for S1P formation, observed by immunofluorescence. Confirming our previous reports, this result strongly supports the hypothesis about the anti-tumoral mechanism of action of luteolin.

An interesting outcome came from the autophagy and apoptosis assays. Autophagy is a physiological biologic process for the degradation and elimination of damaged organelles and misfolded proteins, acting in response to cell death, development, starvation and tumor suppression. Normally, cells exploit basic autophagy to maintain the biological function, homeostasis and control of cell content. In stem cells, autophagy is associated with the maintenance of peculiar properties as self-renewal and differentiation. Therefore, in cancer stem cells, autophagy facilitates tumorigenesis promoting cell survival, tumor growth and progression. Noteworthy, autophagy in cancer may serve also as a tumor suppressor mechanism, by oxidative stress induction, nutrient deprivation and degradation of damaged organelles [20]. However, in extreme hypoxic condition as in GBM, autophagy can be reasonably utilized to overcome for various stress and fulfill the high metabolic demand, thus enhancing tumor progression and survival. 

From this view, the reported ability of luteolin to decrease the autophagy process in GSCs seems noteworthy, as it can mean the disruption of the stemness properties influence on GBM behavior. In parallel, luteolin proved to increase GSC apoptosis as by annexin V assay, a significant increase in phosphatidylserine exposure on the outer layer of the plasma membrane of luteolin-treated cells was shown. The induction of pro-apoptotic mechanisms was confirmed by the upregulation of Caspase-3/-7, known to be effector caspases during cell death cascade, as well as that of Bax, a major pro-apoptotic member of the Bcl-2 family, a central cell death regulator and an imperative gateway to mitochondrial dysfunction.

The gene and protein expression profiling conducted to investigate the molecular mechanisms underlying luteolin effects on GSCs revealed a peculiar pattern characterized also by the downregulation of the proliferative axis of MAPK, Ras/Mek/Erk and PI3K/Akt/mTOR. The MAPK pathway is one of the most ubiquitous and characterized signal transduction axis, acting in response to intrinsic and external stimuli.

MAPK mediators regulate invasive and proliferative cell phenotype, driving the formation and growth of metastases, as well as the invasiveness of tumors to the detriment of surrounding tissues [21]. About 88% of gliomas display alterations in the MAPK pathway, so that high p-MAPK (the active forms) expression is now considered an unfavorable prognostic marker for overall survival and often responsible for increased resistance to therapy in patients with GBM [22].

MAPK signaling plays a crucial role in the onset, progression and relapse of gliomas, so that many tentative single-molecule therapies have been developed targeting, but without any significant advancement in the treatment of these tumors. The reason why may be found in the activation of collateral pathways able to overcome MAPK shutdown, suggesting the necessity to develop and combined treatment strategies acting on multiple foreheads.

For example, the inhibition of the MEK/ERK pathway leads to activation of PI3K/mTOR signaling, but the combined targeting proved to reduce self-renewal and tumorigenic capacity of glioma stem cells [23]. The PI3K/Akt/mTOR signaling is constitutively activated in most GBMs, resulting in the constant activation of prosurvival signals, with the blocking of tumor suppression pathways, so that the inhibition of these two pathways in conjunction with p53 activation may have the ability to induce cell death in GBMs and differentiation of GSCs, as previously suggested by Daniele et al. [24].

The two impacted pathways that mostly deserve consideration are certainly those regulating cell cycle and sphingolipid rheostat. From gene expression, in fact, emerged an upregulation of the most characterized cell cycle controllers p53 and p27, in parallel with the downexpression of cyclins and kinases.

The processes of the cell cycle, as the passages through cycle phases, are controlled by two groups of regulatory proteins, cyclins and cyclin-dependent kinases (CDKs). The synergic activity of these two families of proteins triggers a cascade of phosphorylation resulting in the transcription of factors encoding for DNA replication signals and access in the S phase of cell cycle. Upon irreversible damage or stress signals, both p53 and p27 are known to act by depleting regulators of cell cycle progression, such as cyclins (A, B1, B2, D) or *CDKs* (*CDK1, -2, -4, -6*), inducing the formation of suppressor complexes and then inducing apoptosis [25].

The most common alterations found in GBM affect p53 [26], with loss or mutation of the p53 gene, promoter methylation, which results in p53 inactivation, with impairment of p53 stability, and activity suppression through amplification of p53 inhibitor genes. 

From these premises, the overexpression of p53 and p27, together with the reported downregulation of *CDK1, CCNB1* and *CCND1*, seems really promising for the development of an add-on therapy able to block GSC cycle progression and inducing apoptosis.

Interestingly enough, protein expression analysis revealed that luteolin was able to decrease the level of MGMT. 

It is important to evoke that the examined GSC samples were selected for the hypomethylation of the *MGMT* gene promoter, with consequent upregulation of its expression and activity. The activity of *MGMT* consists in repairing DNA damage found during cell replication, including damage created by the action of chemotherapeutic drugs used as anticancer agents, which should block tumor growth. In doing so, the high expression of *MGMT* in hypomethylated patients determines the reversion of the activity of the chemotherapy, making patients resistant to chemotherapy, and this is why, on the contrary, patients with a methylation level >9% have a better prognosis. From these premises, the ability of luteolin to reverse this epigenetic mechanism, decreasing the expression of *MGMT* despite the hypomethylation of its promoter, appears truly promising.

**Figure 9:**
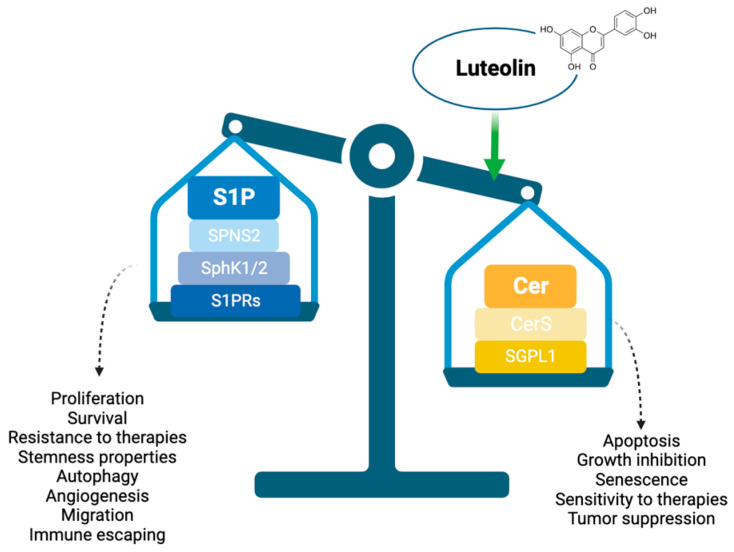
Graphic representation of the action of luteolin in shifting the balance of sphingolipid rheostat toward the proapoptotic Ceramide, with the consequent increase of apoptosis, chemosensitivity and tumor suppression.

Finally, data confirming our starting hypothesis proved that luteolin determines a shift in the balance between S1P and ceramide, in favor of ceramide (Figure 9), by inducing the downregulation of both *SPHK1/2*, and of the S1P transporter outside cells, *SPNS2*. These events were paralleled by the increase of *SGPL1*, the lyase responsible for S1P catabolism and *CERS1*, the enzyme responsible for ceramide formation. Notably, the contrary overexpression of S1P receptor 1–3 may be justified by the tentative recall of extracellular S1P, in an attempt of cells to resist and recover. 

S1P and Ceramide are essential sphingolipids with opposite effects on cell fate, especially in tumors, where cell proliferation is extremely overrepresented.

While ceramide inhibits tumor cell growth and proliferation by inducing apoptosis, S1P acts to promote cell survival, proliferation and even nutrient and oxygen supply by angiogenic mechanisms. For these reasons, their delicate balance both at intra- and extracellular level is critical for tumor progression and therapy resistance. Precisely, in cancer, this equilibrium may be disrupted, with the consequent alteration of bioactive sphingolipid levels, so that they have the potentiality to act as tissue and circulating biomarkers of cancer progression, metastasis and relapses. 

Increasing data report the potential function of S1P as cancer biomarker, since its altered levels in plasma of cancer patients. For example, ovarian cancer patients present two-fold higher S1P levels than healthy controls, and similarly, high S1P plasma levels have been associated with an increased risk of developing lung cancer [27,28]. In prostate cancer, S1P proved to be correlated with prostate-specific antigen (PSA) and lympho node status and the same authors hypothesized that S1P and Sphk1 transported inside red blood cells, the major biological source of blood S1P, may be considered reliable biomarkers for the early detection of prostate cancer [29]. A Japanese study reported that S1P measured by LC-ESI-MS/MS in breast cancer tissues was higher than in healthy ones, suggesting not only a role of S1P in tumor onset and progression but also in the interaction with tumor microenvironment [30].

At a cellular level, many protumor signal pathways are finely regulated by S1P, being implicated in inflammation and cancer. In A549 lung adenocarcinoma cells, S1P is able to promote the production of cycloxygenase2 (COX2) and prostaglandin E2 (PGE2) and the TNFα/IL1β-induced activation of SphK1 is crucial for cancer cell survival and inflammatory boost [31,32]. The use of a SphK1−/− mouse model proved that SphK1-produced S1P promotes pancreatic cancer progression [33]. Similarly, mice lacking S1P lyase at the intestinal level exhibited greater disease activity of colitis-associated cancer, cytokine level increase, S1P accumulation, tumor formation, colon shortening and STAT3 activation [34].

The other face of the coin is represented by ceramide, which acts inducing apoptosis by changing the mitochondrial ultrastructure, thus reducing mitochondrial function and membrane potential. Notably, the different length of ceramide chains affects the biological functions in vivo. C18-Cer, for example, is able to modulate telomerase activity and mitochondrial-induced cancer cell apoptosis in head and neck squamous cell carcinoma [35], while C16-Cer is able to promote ER stress-mediated apoptosis in lung cancer [36]. In colon cancer, ceramide blocks cell proliferation and migration through the downregulation of IL-10, STAT3 and NF-kB expression [37]. Similarly, the overexpression of C16-Cer in breast cancer reduces the phosphorylation of ERK and Akt/mTOR, in turn reducing cell proliferation and survival [38].

Particularly in GBM, S1P has been implicated in many aggressive tumor phenotypes, as patient samples are characterized by the rheostat shift toward S1P against ceramide [39]. Abuhusain et al. reported that C18 ceramide suffers a dramatic decrease compared to C24:1 and C16 [39]. The same authors demonstrated also that S1P concentration are positively associated with tumor grade and determines an angiogenic boost. S1P proved to contribute to GBM invasion with a mechanism involving the upregulation of urokinase plasminogen activator [40]. The upregulation of S1P observed in GBM is closely related to the upregulation of the enzymes responsible for S1P production. In this context, the acid ceramidase (acid CDase) and SphK1, which drive rheostat from ceramide to S1P, were reported to be higher in GBM tissues compared to normal brain [39], with acid CDase associated also to neural stem cell-like brain tumor-initiating cells (BTIC) markers [41]. We also report for the first time that S1P is able to act as a proliferative and pro-stemness autocrine factor for GSCs, promoting both their cell cycle progression and stemness phenotypic profile [18]. We further demonstrated that plasma membrane enrichment of SphK 2 in brain endothelial cells leads to increased cellular level of S1P, and significant potentiation of its secretion. In turn, extracellular S1P stimulates GBM cell proliferation, and brain endothelial cell migration and angiogenesis [42].

From these premises, it appears scientifically sound that inducing the shift in the rheostat toward the production of Ceramide against S1P may revert the permissive contribution of TME on tumor progression and resistance, with an enormous advantage on clinical outcome. 

Aside the pharmacologic compounds known to interfere with S1P signaling, as Fingolimod (FTY720, Gilenya, Novartis) used for the treatment of relapsing–remitting multiple sclerosis [43], a first-in-class S1P receptor modulator, several natural compounds have been described to impact sphingolipid rheostat [44]. Among them, luteolin deserves consideration due to its proved ability to influence cancer behavior. 

Luteolin, in fact, exhibited anticancer activity on many tumor subtypes, interfering with different molecular mechanisms. In colon cancer, luteolin exerts an anti-inflammatory and antioxidant ability, decreasing the expression of inducible nitric oxide synthase (iNOS) and cyclooxygenase-2 (COX-2) [45]. Luteolin further determines the suppression of matrix metalloproteinase-2 (MMP-2) and MMP-9, thus inhibiting angiogenesis an invasion, two key mechanisms of tumor progression and metastasis [46]. Additionally, Pandurangan et al. reported that luteolin promotes the expression of nuclear factor erythroid 2-related factor 2 (Nrf2), recognized as a crucial transcription factor with anticarcinogenic properties related to the Nrf2/antioxidant responsive element (ARE) pathway [47].

In lung cancer, luteolin confirms its ability to interfere with cancer progression and development, by inhibiting migration of NSCLC cells and inducing apoptosis, through the incremented activation of Caspase-3 and Caspase-9, decreased Bcl-2 and increased Bax expressions [48]. According to our results, in 2013, Park et al. proved that luteolin could also induce autophagy, and that the inhibition of autophagy determined a reduction of apoptotic cell death, suggesting that Luteolin-induced autophagy functions as a cell death mechanism [49].

In GBM, the available scientific works have mostly been carried out on commercial cell lines, which makes our results even more significant and promising. However, luteolin was found to reduce viability of A172 and U-373MG glioblastoma cells in a dose- and time-dependent manner, with concentrations higher than 100 µM causing morphological change related to apoptosis and nuclear fragmentation [50]. Furthermore, luteolin demonstrated efficacy in reducing tumorigenesis and migration of U-251 cells, with parallel phosphorylation of ERK proteins, cleavage of PARP and Caspase-9, promotion of DNA damage and depolarization of mitochondrial membrane [51].

All of these findings support the thesis that interfering with sphingolipid rheostat by specific targeting in favor of ceramide has the potential to induce tumor cell death, as a direct effect and by reducing drug resistance. In addition, the targeting with a natural compound represents an added value in terms of add-on therapy, minimizing the potential side effects occurring with a multiple pharmacological therapeutic strategy.

## 4. Materials and Methods

### 4.1. Study Design

For this study, n = 10 tumors of patients operated at the Neurosurgery Unit of Fondazione IRCCS Ca’ Granda Ospedale Maggiore Policlinico in Milan (Italy) were selected from our biobank and processed to isolate cell subpopulation. The Institutional Review Board approved the protocol (IRB#1670/2015) and all patients provided a signed informed consent. Inclusion criteria were: (1) signed consent for the study; (2) histologically proven diagnosis of GBM according to the WHO classification (2016) on review by two independent pathologists; (3) age between 18 and 80 years; (4) Karnofsky Performance Status (KPS) > 60; (5) MGMT promoter methylation value < 9% [6].

### 4.2. Tumor Processing and Cell Culture

GBM samples directly taken from the operating room were transferred to the Laboratory of Experimental Neurosurgery and Cell Therapy of the Ospedale Maggiore Policlinico, where a tissue portion was subjected to mechanical digestion first and enzymatic digestion in solution with trypsin 0.25% 2 mL (ThermoFisher Scientific, Waltham, MA, USA, incubated for one hour at 37 °C) next. Once dissolution of the extracellular matrix was achieved, the solution containing the cells with trypsin was diluted in 5 mL of Dulbecco’s Modified Eagle Medium/Nutrient Mixture F12 solution (DMEM/F12, ThermoFisher Scientific, Waltham, MA, USA) supplemented with 10% Fetal Bovine Serum (FBS, Euroclone, Milan, Italy) to inhibit trypsin. Next, the solution was filtered through a 70 µm filter to separate the GBM cells from the remaining matrix and finally centrifuged to settle on the bottom of the falcon. The cells in the pellet were withdrawn and placed in a 25 cm^2^ flask containing Human Medium, a specifically formulated medium to isolate GSCs, as previously described [18], consisting of Dulbecco’s modified Eagle’s medium (DMEM)/F12 (1:1) containing 10 ng/mL fibroblast growth factor-2 (bFGF, Peprotech Inc. Rocky Hill, NY, USA), 20 ng/mL epidermal growth factor (EGF, R&D Systems, Minneapolis, MN, USA) and 1% penicillin/streptomycin (ThermoFisher Scientific, Waltham, MA, USA). Finally, the flask was left in an incubator at 37 °C in hypoxic condition (5% O_2_) with 5% CO_2_, to recreate the in vivo microenvironment. Notably, this protocol, already validated, allows the isolation and culturing of primary GBM-derived GSCs, growing as floating neurospheres and expressing tumor stem cell markers [18].

### 4.3. Cell Counting

During the experiments, cell counts were performed to monitor cell proliferation and cell healthy status. GSCs were aspirated by successive washes with a few mL of Phosphate-Buffered Saline (PBS, Euroclone, Milan, Italy), diluted with Trypan Blue (ThermoFisher Scientific, Waltham, MA, USA) and placed on the Fuchs-Rosenthal counting chamber. The ratio of live to dead cells was also calculated, due to the penetration property of Trypan Blue through the plasma membrane, which causes the dead cells to take on a blue tint.

### 4.4. Pyrosequencing Analyses

The Therascreen MGMT Pyro Kit and the PyroMark Q24 system (both from Qiagen, Milan, Italy) were used to assess the methylation status of the MGMT gene promoter. In brief, bisulfite-converted genomic DNA was amplified by PCR, the amplicons were immobilized on streptavidin beads and single-stranded DNA was prepared, sequenced and finally analyzed on the PyroMark Q24 system.

### 4.5. Drug Administration

The degree of TMZ resistance, as well the effects of S1P and luteolin were measured by the administration of increasing doses to GSC culture media. In particular, TMZ (100 μM, 250 μM and 500 μM, Selleck Chemical, Berlin, Germany), S1P 200 nM ((Enzo Life Sciences, Farmingdale, NY, USA) and luteolin (10 μM, 20 μM and 50 μM, Sigma Aldrich, St. Louis, MO, USA) were added for 72 h to cell culture and removed before further cellular and molecular analysis. The same challenging with luteolin was performed on commercial lines of healthy brain-derived cells, i.e., astrocytes, neuronal primary cells and neuronal progenitor stem cells, considered as normal brain cell counterparts. Astrocytes were purchased by ABM (Cat. N. T0280), while NPC and NPSC were purchased by CelProgen (Cat. N.: 36057-01 and 36057-02).

### 4.6. MTT Assay

GSCs (n = 5 × 10^3^) were plated into 96-well plates and treated with the desired compounds for 72 h. At the end of treatment, cells were placed in DMEM/F12 containing (3-(4,5-Dimethyl-2-thiazolyl)-2,5-diphenyl-2H-tetrazolium bromide (MTT, Sigma Aldrich, St. Louis, MO, USA) solution and then incubated for 4 h at 37 °C. After that, the solution with MTT was aspirated and replaced with Lysis Buffer (2-isopropanol 95%, formic acid 5%). The plate was shaken for 10 min at speed 50 and finally analyzed in the Synergy H1 Microplate Reader (BioTek, Agilent, Milan, Italy).

### 4.7. Immunofluorescence Assay

GSCs were seeded on 96-well imaging cell plates, treated and left to incubate at 37 °C in hypoxia for 48 h. Then cells were fixed with 8% paraformaldehyde and incubated at 37 °C for 30 min. Once the medium was aspirated with the fixative, a wash with PBS was performed. The plate was then left at 4 °C overnight with 100 µL of PBS per well. After 24 h, the plate was incubated with 0.1M 50 µL glycine for 10 min. After two washes with PBS, permeabilization with PBS + Triton 0.5% was performed. Before applying the primary antibody, Blocking Buffer PBS + BSA 5% was added for 30 min, and finally, primary antibodies were diluted in blocking buffer 1:200. The next day, the primary antibodies (Table in below) were removed, and after three washes with PBS, secondary antibodies diluted in Blocking Buffer at a concentration of 1:500 were added and left to incubate in the dark for 45 min at room temperature. Once the secondary antibodies were removed, DAPI diluted in Blocking Buffer 1:500 was finally added and incubated for 30 min in the dark at room temperature and then removed with three washes in PBS.

### 4.8. Autophagy Assay

To assess the activation ability of autophagy mechanisms, GSCs were plated, treated and then incubated for 20 min at room temperature with acridine orange 1:1000 and Hoechst 1:1000 (Sigma Aldrich, St. Louis, MO, USA). After three washes in PBS, the cells were fixed with 4% paraformaldehyde and finally analyzed with a microplate reader.

### 4.9. Annexin V Apoptosis and Necrosis Assay

To study the degree of apoptosis induced by the treatments administered to the cells, CaCl2, Necrosis Detection Reagent, Annexin NanoBiT^®^ Substrate, Annexin V-SmBiT and Annexin V-LgBiT at a 500-fold dilution were added to the medium purchased by RealTime-Glo™ Promega (Promega Italia, Milan, Italy). Luminescence was analyzed with a microplate reader after 8 h.

### 4.10. RNA Extraction and Quantification

RNA extraction was performed with TRIzol (ThermoFisher Scientific, Waltham, MA, USA), to which chloroform (0.3 mL/mL TRIzol used) was added to separate the three phases (RNA, DNA and protein). The aqueous phase containing the mRNAs was aspirated and placed on another tube with 0.5 mL/mL TRIzol used of 2-propanol and 2 µL of glycogen. After centrifuging and reaspirating the supernatant, 0.3 mL of 75% ethanol per mL of TRIzol used and finally 20 µL of sterile water was added. The final RNA concentration was quantified by NanoDrop Spectrophotometer (ThermoFisher).

### 4.11. Real-Time Quantitative Reverse Trascription PCR

Freshly quantified RNA was retrotranscribed according to the TranScriba Cabru protocol (1 μg). Complementary DNA (cDNA, 1 μg) was plated for qRT-PCR together with specific primers and SYBR Green qPCR Master Mix (ThermoFisher Scientific, Waltham, MA, USA) r). The StepOne Real-Time PCR System (ThermoFisher Scientific, Waltham, MA, USA) thermal cycler was used for cDNA amplification with the following steps: (1) initial polymerase activation at 95 °C for 12 min (1 cycle); (2) denaturation at 95 °C for 15 s, annealing at 62 °C for 20 s and elongation at 72 °C for 20 s (40 cycles). Data were normalized to 18S expression, used as endogenous control. Relative gene expression was determined using the 2−ΔΔCt method. Specific primer sequences and relative melting temperatures are listed in Table 1.

### 4.12. Western Blot Analyses

After treatment, GSCs were lysed with M-PER Protein Extraction Reagent (ThermoFisher Scientific, Waltham, MA, USA) in presence of Halt Protease Inhibitor Cocktail (ThermoFisher Scientific, Waltham, MA, USA). Proteins were quantified by the Pierce Detergent Compatible Bradford Assay Kit (ThermoFisher Scientific, Waltham, MA, USA). Protein lysates (20 μg) were separated in Bolt 10% Bis-Tris Plus Gels (ThermoFisher Scientific, Waltham, MA, USA) in a Mini Gel Tank (ThermoFisher Scientific, Waltham, MA, USA) and transferred into nitrocellulose iBlot 2 Transfer Stacks using iBlot 2 Dry Blotting System (ThermoFisher Scientific, Waltham, MA, USA). After transfer, the membrane was blocked in Tris-buffered saline/Tween 20 þ 5% milk solution and incubated separately with the indicated primary antibodies, overnight at 4 °C (Table 2). After incubation with HRP-labeled secondary antibody (Invitrogen, Carlsbad, CA, USA), protein bands were scanned with SuperSignal West Pico PLUS Chemiluminescent Substrate (ThermoFisher Scientific, Waltham, MA, USA) and detected by ChemiDoc XRSþ (Bio-Rad, Hercules, CA, USA). Densitometric analyses were performed using ImageJ (https://imagej.net/ij/download.html).

### 4.13. Statistical Analyses

The IBM SPSS Statistics 28.0 software was used to calculate individual means, group mean and standard deviation of the mean. Additionally, t-student correlation analysis was utilized. Differences were considered statistically significant for *p* < 0.05. Values are expressed as mean ± SD of at least three independent experiments.

## 5. Conclusions

The knowledge about the contribution of altered sphingolipid metabolism to carcinogenesis, cancer progression and chemotherapeutic resistance is well known, giving the rheostat a crucial role in the regulation of cell fate. Our results show that sphingolipid rheostat represents an additional pathogenetic mechanism exploited by glioblastoma to proliferate and survive treatments, and that luteolin may be considered a viable therapeutic option to be considered for patients with GBM who respond poorly to first-line treatments. These results are in line with the approach of molecular target-therapy, and they can be used as a tool to screen and cluster patients based on expression profile, with the aim to apply a precision medicine approach.

## Figures and Tables

**Table 1:** List of primers used for qRT-PCR, with sequences and melting temperature.

Gene	Forward Primer (5′-3′)	Reverse Primer (3′-5′)	T_m_, °C
*18S*	ACTTTCGATGGTAGTCGCCGT	CCTTGGATGTGGTAGCCGTTT	61
*RAS*	AGCAGGTGGTCATTGATGGG	CCGTTTGATCTGCTCCCTGT	60
*MEK-1*	CTTCGCAGAGCGGCTAGG	AGCTCTAGCTCCTCCAGCTT	61
*ERK-1*	ACTCCAAAGCCCTTGACCTG	CTTCAGCCGCTCCTTAGGTA	60
*PI3K*	GCTCCTAGCAGAAGCCTATG	TCTGGTCCTCCCGGTACA	60
*AKT*	TCTATGGCGCTGAGATTGTG	CTTAATGTGCCCGTCCTTGT	58
*mTOR*	CTTAGAGGACAGCGGGGAAG	TCCAAGCATCTTGCCCTGAG	62
*MDR1*	CCCATCATTGCAATAGCAGG	GTTCAAACTTCTGCTCCTGA	57
*P53*	AGGCCTTGGAACTCAAGGAT	CCCTTTTTGGACTTCAGGTG	58
*P27*	TGGCTTGTCAGGAACTCGAC	CTAGTCTCCAGGGAGGTGCT	63
*CDK1*	AAACTACAGGTCAAGTGGTAGCC	TCCTGCATAAGCACATCCTGA	61
*CCND1*	TGGAGCCCGTGAAAAAGAGC	TCTCCTTCATCTTAGAGGCCAC	61
*CCNB1*	ACTGGGTCGGGAAGTCACTG	CATTCTTAGCCAGGTGCTGC	61
*CASPASE-3*	ATGGTTTGAGCCTGAGCAGA	GGCAGCATCATCCACACATAC	60
*CASPASE-7*	GAGCAGGGGGTTGAGGATTC	GTCTTTTCCGTGCTCCTCCA	61
*CASPASE-9*	GCAGGCTCTGGATCTCGGC	GCTGCTTGCCTGTTAGTTCGC	63
*BAX*	AGCAAACTGGTGCTCAAGG	TCTTGGATCCAGCCCAAC	57
*BCL-2*	AGTACCTGAACCGGCACCT	GCCGTACAGTTCCACAAAGG	60
*SPHK1*	TGCAGTTGGTCAGGAGGTCT	GCTCTGGTGGTCATGTCTGG	66
*SPHK2*	CCCCGGTTGCTTCTATTGGT	ATCCCACTCACTCAGGCTCA	66
*SPNS2*	GCAGCTACGTCTTCTCCTCC	AGGTGATGGCCCCAAAGATG	67
*S1PR1*	GGGAGCAATAACTTCCGCCT	AAGCAGAGTGAAGACCGTGG	66
*S1PR3*	CAACCACAACAACTCGGAGC	GCCAACACGATGAACCACTG	64
*SGPL1*	AAGCATATCGGGATCTGGCC	TAGCTCTTCTCATTGCCCGC	65
*CERS1*	CCCTTCTTCCATGACCCACC	CTCAGTGGCTTCTCGGCTTT	61

**Table 2:** List of specific antibodies used for IF and WB, with relative supplier and concentration tested.

Antibody	Supplier	Catalog Number	Concentration
β-actin	Sigma, St. Louis, MO, USA)	A5441	1:500
RAS	SantaCruz Biotechnology (Dallas, TX, USA)	Sc-35	1:500
MEK	SantaCruz Biotechnology (Dallas, TX, USA)	Sc-81504	1:500
ERK-1	(ThermoFisher Scientific, Waltham, MA, USA)	44-654G	1:1000
ERK-2	(ThermoFisher Scientific, Waltham, MA, USA)	44-654G	1:1000
CASP3	Cell Signaling Technology (Danvers, MA, USA)	9662	1:1000
CASP7	SantaCruz Biotechnology (Dallas, TX, USA)	Sc-56063	1:500
SPHK1	Cell Signaling Technology (Danvers, MA, USA)	3297	1:1000
SPHK2	Abcam (Cambridge, UK)	Ab37977	1:1000
SGPL1	Abnova (Taipei, Taiwan)	H00008879-W01P	1:500
MGMT	SantaCruz Biotechnology (Dallas, TX, USA)	Sc-166528	1:500

## Data Availability

Data are contained within the article.
