# Correction: Navone et al. Role of Luteolin as Potential New Therapeutic Option for Patients with Glioblastoma Through Regulation of Sphingolipid Rheostat. *Int. J. Mol. Sci.* 2024, *25*, 130

**DOI:** 10.3390/ijms27104293

**Published:** 2026-05-12

**Authors:** Stefania Elena Navone, Laura Guarnaccia, Massimiliano D. Rizzaro, Laura Begani, Emanuela Barilla, Giovanni Alotta, Emanuele Garzia, Manuela Caroli, Antonella Ampollini, Aniello Violetti, Noreen Gervasi, Rolando Campanella, Laura Riboni, Marco Locatelli, Giovanni Marfia

**Affiliations:** 1 Laboratory of Experimental Neurosurgery and Cell Therapy, Neurosurgery Unit, Foundation IRCCS Ca’ Granda Ospedale Maggiore Policlinico, 20122 Milan, Italy; stefania.navone@policlinico.mi.it (S.E.N.); laura.guarnaccia@policlinico.mi.it (L.G.); laura.begani@policlinico.mi.it (L.B.); manuela.caroli@policlinico.mi.it (M.C.); antonella.ampollini@policlinico.mi.it (A.A.); marco.locatelli@policlinico.mi.it (M.L.); 2 Andremacon Biotech Srl, Viale Ortles, 22/4, 20141 Milan, Italy; e.barilla@andremacon.com (E.B.); g.alotta@andremacon.com (G.A.); campanella.rolando@gmail.com (R.C.); l.riboni@andremacon.com (L.R.); 3 Reproductive Medicine Unit, Department of Mother and Child, San Paolo Hospital Medical School, ASST Santi Paolo e Carlo, 20142 Milan, Italy; emanuele.garzia@asst-santipaolocarlo.it; 4 Aerospace Medicine Institute “A. Mosso”, Italian Air Force, 20138 Milan, Italy; 5 Space Attache’, Embassy of Italy in Washington DC, Washington, DC 20008, USA; aniello.violetti@esteri.it; 6 Alcamena Stem Cell Therapeutics, 1450 South Rolling Road, Suite 4.069, Halethorpe, MD 21227, USA; 7 Department of Medical-Surgical Physiopathology and Transplantation, University of Milan, 20122 Milan, Italy

In the original publication [1], there was a mistake in Figure 1A as published. In the original version of Figure 1A, the image for the TMZ 250 μM treatment has an unintentional graphical error. A microphotograph from the TMZ 100 μM treatment was inadvertently inserted in the TMZ 250 μM treatment group during final layout assembly.

The corrected Figure 1 (attached) now accurately reflects the “Representative images of GSC challenged with increasing doses of TMZ”. The data on cell viability of Figure 1B remains unchanged. The corrected Figure 1A appears below. The authors state that the scientific conclusions are unaffected. This correction was approved by the Academic Editor. The original publication has also been updated.

## Figures and Tables

**Figure 1:**
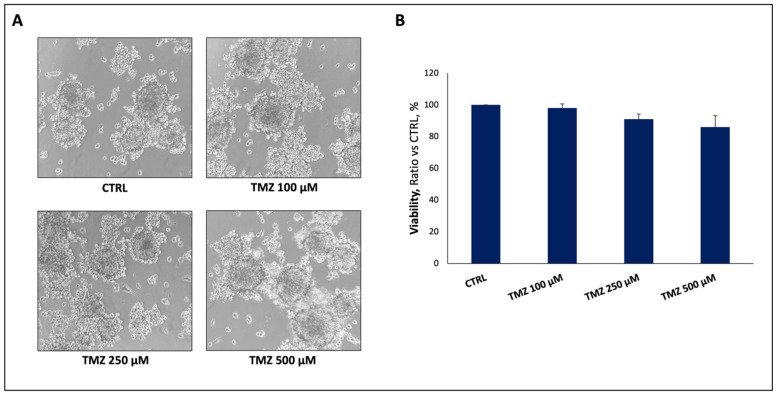
(**A**) Representative images of GSC challenged with increasing doses of TMZ, reporting a dose-independent chemotherapy resistance. (**B**) Viability data of GSC treated with increasing doses of TMZ. No statistical significance was reported in the treatment groups compared to control. Data are the means ± standard deviation of at least three experiments, run in triplicate. Magnification 10×.
